# Effects of a maternal role adjustment program for first time mothers who use postpartum care centers (*Sanhujoriwon*) in South Korea: a quasi-experimental study

**DOI:** 10.1186/s12884-020-02923-x

**Published:** 2020-04-16

**Authors:** Ju-Eun Song, Hyun-Ju Chae, Jung Mi Ko, Jeong In Yang, Tiffany Kim

**Affiliations:** 1 College of Nursing・Research Institute of Nursing Science, Ajou University, Suwon, Republic of Korea; 2 Department of Nursing, Joongbu University, 201, Daehak-ro, Chubu-myeon, Geumsan-gun, Chungnam, 32713 Republic of Korea; 3 Department of Nursing, Kookje University, Pyeongtaek, Republic of Korea; 4 College of Nursing, Ajou University, Suwon, Republic of Korea; 5 College of Medicine, Ajou University, Suwon, Republic of Korea; 6 School of Nursing, Bouvé College of Health Sciences, Northeastern University, Boston, USA

**Keywords:** Mothers, Maternal behavior, Breastfeeding, Ecology

## Abstract

**Background:**

Many South Korean women stay in specialized postpartum care centers called *Sanhujoriwon* for 2 weeks after childbirth, a time which is widely recognized as a critical period for maternal role adjustment. Mothers’ time within the postpartum care center offers a unique opportunity for nursing intervention to promote a successful transition to motherhood, especially for first time mothers. This study aimed to develop a maternal role adjustment program within the *Sanhujoriwon* based on the ecological model, and to evaluate its effects on maternal role confidence and breastfeeding success.

**Methods:**

A non-equivalent control group pretest-posttest design was used. Data were collected from 30 participants in the experimental group and 37 in the control group at four measurement times, i.e., admission day to *Sanhujoriwon*, discharge day from *Sanhujoriwon*, 4–6 weeks postpartum, and 12 weeks postpartum. The experimental group received the maternal role adjustment program, which included family education and counseling regarding breastfeeding and infant care, and encouraged rooming-in practices during their stay in the *Sanhujoriwon*. The data were analyzed using the IBM SPSS statistics 25.0 program using descriptive statistics, t-test, chi-square test, ANCOVA, and GEE.

**Results:**

There were significant interaction effects showing different patterns in maternal role confidence and breastfeeding success scores over the four time points. Maternal role confidence in the experimental group gradually increased over time. Maternal role confidence in the control group also increased from baseline to 4 to 6 week postpartum, but abruptly decreased at 12 week postpartum. At 12 weeks postpartum, maternal role confidence in the experimental group was significantly higher than that of the control group. In addition, breastfeeding success scores in the experimental group also gradually increased over the four time points, while those of the control group showed a gradual decline. Breastfeeding success scores were significantly higher than those of control group at both 4–6 weeks and 12 weeks postpartum.

**Conclusions:**

These results indicate that the maternal role adjustment program was effective in improving maternal role confidence and breastfeeding success among first time mothers in the postpartum care center.

## Background

The postpartum period is a time of great vulnerability for many women [1]. Throughout the world, many culturally-specific traditional care practices for postpartum women are observed to ensure recovery and avoid health problems in later years [2].

In South Korea, postpartum women were traditionally provided with care in homes called *Sanhujori* for 2–3 weeks following delivery by non-professional caregivers such as their mother or mother-in-law [3]. But recent societal changes to the extended nuclear family system and increased postpartum care needs have made traditional *Sanhujori* in homes difficult to maintain. This has driven the emergence of stand-alone postpartum care center called *Sanhujoriwon* [4]. These are nonmedical commercial facilities, where women and infants stay for a couple of weeks right after discharge from the hospital. *Sanhujoriwon* first emerged in 1996 and their numbers and utilization have dramatically increased since that time [5]. In 2018, a total of 584 of these facilities were in operation [5]. According to recent Korean national statistic, 75% of delivering women in South Korea used *Sanhujoriwon* in 2018 [6]. *Sanhujoriwon* continue be an important part of postpartum care for women and their infants in South Korea and are only expected to increase in number in the coming years [4–6].

However, it appears that *Sanhujoriwon* have some significant problems in maternity care management, particularly related to breastfeeding success, family-infant bonding and overall infant care ability. Although childbirth and parenthood is a family-centered phenomenon and family centered care is widely recognized as the global standard of care during the postpartum period [7–9], rooming-in is not currently the norm within *Sanhujoriwon* [6, 10]. Not surprisingly, this environment is associated with decreased breastfeeding success and increased parental stress after discharge from *Sanhujoriwon* [10, 11]. According to a national survey in 2018 regarding *Sanhujoriwon* use, only 3% of postpartum women using *Sanhujoriwon* “roomed in” with their infants for 24 h and the average daily time spent with infants was only 4.2 h [6].

Many mothers view their postpartum stay within the *Sanhujoriwon* as an opportunity for rest and recovery without parenting responsibilities [11]. Therefore many mothers in *Sanhujoriwon* do not desire to room-in and are separated from their babies most of the time [10, 11]. Likewise, most *Sanhujoriwon* engage in rooming-in practices only if mothers specifically request it in order to promote sufficient rest for new mothers [10]. Also *Sanhujoriwon* commonly limit the presence of family members except partners for infection control. Accordingly, partners are the primary source of family support to mothers in the *Sanhujoriwon*, but they usually also need support and education for parenting role adjustment [11]. Therefore, most mothers in *Sanhujoriwon* spend their early postpartum period without sufficient mother-infant interaction and social support from family members to assist in a successful maternal transition to motherhood in the early period after childbirth [10, 11].

Mother-infant interaction and social support are important influencing factors on maternal role adjustment for the first time mother [11, 12]. So lessening these opportunities within the *Sanhujoriwon* may have a negative impact on infant care ability and breastfeeding success of first time mothers. Previous studies to explore the effect of *Sanhujoriwon* use reported that parenting stress and postpartum depression were higher in mothers who used *Sanhujoriwon* than mothers who did not use it after discharge from *Sanhujoriwon* [11, 13]. A qualitative study on experiences of *Sanhujoriwon* use also showed that first time mothers who stayed in *Sanhujoriwon* had more difficulty breastfeeding and providing infant care after discharge [10]. Since the use of *Sanhujoriwon* is expected to continue to rise [4–6], it is imperative that interventions for promoting maternal role adjustment be developed and implemented. It will be especially important to focus these family centered interventions on infant care and breastfeeding during this critical maternal transition period within the *Sanhujoriwon* [10, 11, 13].

Several intervention studies for promoting maternal role adjustment of mothers in *Sanhujoriwon* have been published [14]. Most of these studies provided educational interventions focused on parenting knowledge and skill to mothers without partners and didn’t fully consider the family unit approach or the environmental characteristics of the *Sanhujoriwon* [15–17].

Bronfenbrenner’s ecological system model [18] provides a robust theoretical foundation for the family unit approach within the environment of the *Sanhujoriwon,* as it allows for inclusion of a multitude of environmental factors surrounding individuals. The ecological model can help explain human behavior and development [19] and has been used in previous studies to explain parenting stress or development [20–22]. The ecological model posits that an individual’s development is influenced by various environment system surrounding individuals [18, 23]. According to the ecological model, the mother’s development or behavior is not only affected by her own characteristics (the individual level), but also by the various settings of her immediate environment (the microsystem level), such as partner and her baby, and by the interrelationships amongst the settings of her immediate environments (the mesosystem level). The mother’s behavior is further influenced by the broader social setting (the exosystem level), such as family centered care system and care principles for family supports in the *Sanhujoriwon*. These, in turn, are influenced by cultural value or belief toward family centered care practices in the *Sanhujoriwon* (the macrosystem level) [18]. A mother’s adjustment could be enhanced by maximizing the healthy effects of all the various systems within the ecological model.

To our knowledge, this is the first intervention study focused on maternal role adjustment for first time mothers using *Sanhujoriwon* based on the ecological model. In this paper, we present a family-centered care education and counseling intervention encouraging infant care practices and breastfeeding while rooming-in with mothers and partners, and evaluate its effect on maternal role confidence and breastfeeding success.

## Methods

### Design

A non-equivalent control group pretest posttest and repeated measure design was used to compare the effects of maternal role adjustment program for primiparous women who use *Sanhujoriwon* on maternal role confidence for infant care and breastfeeding success. Using a quasi-experimental design, we first recruited participants for the control group and collected their data before implementing the intervention. We then subsequently recruited participants for the experimental group, delivered the intervention, and collected data from them at different time points. Thus randomized sampling can’t be achieved. However, we believe that this time difference allowed us to prevent any diffusion of the intervention between the control and experimental groups.

In this study, data were measured at four times; 2 ~ 7 days after childbirth, i.e., the admission day to *Sanhujoriwon* (baseline), two to 3 weeks after childbirth, i.e., the discharge day from *Sanhujoriwon* (posttest 1), 4 to 6 week postpartum (posttest 2), and 12 week postpartum (posttest 3).

### Participants

All participants were recruited through convenience sampling from two *Sanhujoriwon*. Participants were included if they: (1) were primiparous women who delivered a healthy infant weighing over 2500 g after 37 weeks gestation, (2) were admitted to the *Sanhujoriwon* together with their infants, and (3) voluntarily agreed to participate in this study together with their partner. Exclusion criteria were first time mothers who (1) delivered twins, or (2) had health problems including infection, bleeding, or major depression during childbirth and postpartum period, and needed medical treatments in the *Sanhujoriwon*.

The study sample initially included 40 participants in the control group and 39 participants in the experimental group. In the control group, three participants failed to complete the study because of withdrawal at posttest2 (*n* = 2) and the follow-up loss at posttest3 (*n* = 1). While nine participants from the experimental group failed to finish the study because of loss to follow up at posttest2 (*n* = 3) and posttest3 (*n* = 6). Finally, 37 participants in the control group and 30 mothers in experimental group were included in the final sample, resulting in an attrition rate of 15.2% (12 participants). To check whether the retained sample was biased from the initial sample or not, baseline comparisons were undertaken between mothers lost to follow-up and those retained in the study [24]. There were no significant differences in demographic characteristics such as age, family type, education, occupation, economic status, delivery type, baby gender, and baby’s birth weight between dropouts and the study sample. The retained sample does not appear to differ from those lost to follow up and does not impact our study findings [24].

The total participant number of this study (*N* = 67) exceeds the required minimum sample size (*N* = 24) calculated by the G*power 3.1 program [25] with an alpha of 0.05, power of 0.80, and medium effect size for variance test of 0.25, 2 groups and 4 repeated measurements.

### Intervention

**Fig. 1:**
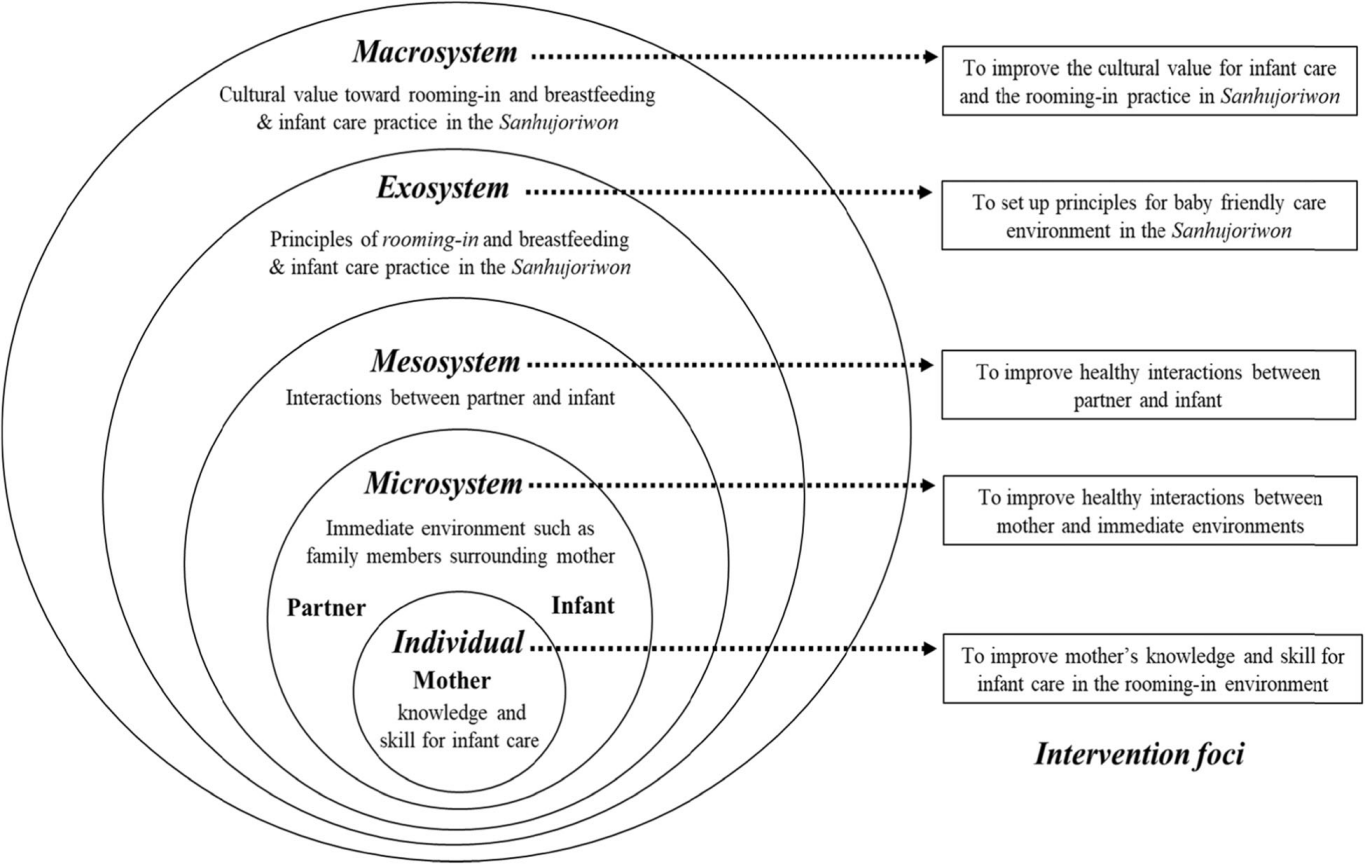
Research framework to show intervention focus in each system for maternal role adjustment program

The maternal role adjustment program was developed based on the ecological model [18] and previous research regarding care needs of postpartum women using *Sanhujoriwon* [10]. The overall research framework for this study is presented in Fig. 1.

According to the ecological model [18], individuals’ behavior is influenced by various multi-level environments surrounding individuals such as the microsystem, mesosystem, exosystem, and macrosystem. Therefore, we considered various factors within each system in the maternal role adjustment program.

For the individual level, group and individual education were provided to mothers. The main goal of this education was to improve mothers’ knowledge and skills regarding infant care and breastfeeding. This education also focused on cultivating a positive attitude related to rooming-in and breastfeeding. Individual counseling and coaching were also provided to facilitate parenting role practice for infant care and breastfeeding, and to address parenting challenges. In the microsystem level, partner education was conducted in order to equip them with improved knowledge and skills in infant care and breastfeeding support. As for the mesosystem level, healthy interactions between partners and their babies were encouraged to help build a more favorable family centered care environment. For the exosystem level, we worked with the *Sanhujoriwon* administrator to encourage and prioritize rooming-in and provide exclusive breastfeeding support in the *Sanhujoriwon*. Lastly, to address the macrosystem level, we tried to change the value regarding postpartum care, especially rooming-in choice and exclusive breastfeeding in the *Sanhujoriwon* through education and counseling. We taught that focusing on mother’s rest with minimal parenting role practice has a negative effect on learning the parenting role, because the early period after childbirth is a very important period for parental role adjustment. Thus, rooming-in to improve interaction between parent and infant, and to create active learning opportunities for parenting role practice is basic and essential to a successful transition to parenthood. The intervention program based on the ecological model is presented in Table 1.

**Table 1:** The maternal role adaptation program based on the ecological model

Ecological system	Meaning	Intervention focus	Intervention goal	Intervention content
Individual	People’s own characteristics such as knowledge, skill, health etc.	First time mothers’ knowledge and skill for infant care in the baby friendly environment	• To improve first time mothers’ core knowledge and skill for infant care and successful breastfeeding practices • To promote mothers’ positive attitudes about rooming-in and breastfeeding practice	• Importance of rooming-in • Successful breastfeeding strategies and breast care • Infant care skills including bathing, diaper changing, playing, interpreting baby’s cues etc.
Microsystem	Immediate environment surrounding individual	Interactions between mother-partner and mother-infant	• To improve the attachment and relationships between mother and infant • To improve the healthy relationship between mother and partner • To promote and empower partners’ support through providing partners core knowledge and skill for infant care and breastfeeding support	• Encourage infant massage by mother • Importance of family centered care and rooming-in • Importance of supporters’ role in successful transition to motherhood • Infant care including bathing, diaper changing, playing, interpreting baby’s cues etc.
Mesosystem	Interactions between individuals in the immediate environment	Interactions between partner and infant	• To improve the attachment and relationships between father and baby	• Encourage infant massage by father • Meaning of baby’s cues and playing with baby
Exosystem	Principles to affect relations and behavior within each environment system	Principles of rooming-in and breastfeeding & infant care support in *Sanhujoriwon*	• To set up desirable principles of rooming-in and exclusive breastfeeding support to establish family centered care environment in *Sanhujoriwon* with Director of *Sanhujoriwon*	• Rooming-in and breastfeeding support and education policies for mother and her family
Macrosystem	Values to affect social interactions that are embedded in each environment system.	Value toward rooming-in and breastfeeding in the *Sanhujoriwon*	• To promote the value of rooming-in and breastfeeding during the early postpartum period as a critical period for maternal role transition and adaptation in *Sanhujoriwon*	• Stress the importance of choosing to room-in and breastfeed to promote a successful transition to mother and father roles

In summary, the intervention was designed to enhance the interactions amongst various support systems and to create a healthy family focused environment to promote infant care and breastfeeding practice. To this end, the authors set up a family-centered rooming-in support system for enhancing parent and infant interactions, and a supportive environment for breastfeeding within the *Sanhujoriwon*. We also encouraged partners to provide positive support for new mothers within the *Sanhujoriwon*. For this purpose, one individual orientation session, two group education sessions, and four individual family counseling & coaching encounters were provided to participants. The intervention was delivered to the participants by 2 members of the research team: the principle investigator (PI), an expert in maternity and women’s health nursing (JES), and a clinical expert in the women’s health (JMK). In order to improve the internal validity of the intervention, JES and JMK met frequently to discuss the study. Details for intervention elements for each session including intervention provider are presented in Table 2.

**Table 2:** Summary of main elements of intervention

When provided	Content	Delivery method	Delivery unit	Delivery setting	Deliverer (Provider)	Exposure quantity	Time span
*Sanhujoriwon* admission day	• Orientation for postpartum care center’s care policies for mother and baby and environment’s characteristics • Orientation for maternal adaptation program and obtaining the written consent form	Explanation Agreement	Individual family (First time mother and her partner)	Maternal private room in *Sanhujoriwon*	Coauthor (JMK)	1 session	30 min
The 1st Saturday after admission into *Sanhujoriwon*	• Core components for enhancing maternal/paternal and baby attachment • Strategies for successful breastfeeding and infant care when rooming-in • Supporter’s role to empower and help the mother’s successful transition • Importance of mothers and fathers’ positive attitudes or values about rooming-in and breastfeeding practice for family-centered care environment	Lecture Practice	Group (First time mothers and their partners)	Lecture room in *Sanhujoriwon*	PI (JES)	1 session	120 min
The 2nd Saturday after admission into *Sanhujoriwon*	• Baby massage and infant care skills • Expression of love among family members, wife, partner, and baby • Supporter’s role and strategies to empower and help mother	Lecture Practice	Group (First time mothers and their partners)	Lecture room in *Sanhujoriwon*	Coauthor (JMK)	1 session	120 min
Twice a week during stay within the *Sanhujoriwon*	• Counseling and individual education for resolving breastfeeding difficulties or infant care problems when rooming-in	Practice Teaching Coaching	Individual family (First time mother and her partner)	Maternal private room in *Sanhujoriwon*	Coauthor (JMK)	4 sessions	30 min for each session
Before starting intervention	• Establishing principles to support breastfeeding and rooming-in for family-centered care environment	Discussion Consensus	Individual (*Sanhujoriwon* administrator)	Meeting room in *Sanhujoriwon*	PI (JES)	1 session	30 min

*PI* Principle Investigator

### Measurements

**Maternal role confidence** was measured using the self-confidence scale developed by Pharis [26]. This scale consisted of 10 items rated on a 5-point Likert scale (1–5), with higher scores indicating higher degrees of maternal confidence for infant care. The internal consistency reliability for this scale at four measurements in this study ranged from .718 to .912.

**Breastfeeding success** was measured by one item with 5 grades about breastfeeding status based on the World Health Organization (WHO) definition of breastfeeding status [27]. This item has been reliably used to measure breastfeeding success in many other studies [28, 29]. According to the WHO, Grade 1 means exclusive breastfeeding, grade 2 means that breastfeeding is much more than formula feeding, grade 3 is the same proportion of breastfeeding and formula feeding, grade 4 means that formula feeding is much more than breast feeding, and grade 5 means exclusive formula feeding [29]. In this study, scores were presented reversed, as suggested by Park [28], so a higher score meant higher level of breastfeeding success. This scale was included in the self-report questionnaire, and sufficient explanation about the meaning of each score was provided to participants.

### Data collection and ethical considerations

Data were collected using a self-report questionnaire from April to August, 2014 at two *Sanhujoriwon* located in one metropolitan city in South Korea. To control the effects of confounding variables such as postpartum care principles for breastfeeding support and infant care [11], two *Sanhujoriwon* having a similar size and care policies for mother and infant were selected for this study. Also, to prevent diffusion of the intervention effect, data collection for the control group was done first, and then data collection for the experimental group was conducted. In this study, data collection for baseline and posttest1 were done at the *Sanhujoriwon,* and data for posttest2 and posttest3 were collected in participant homes through a mail survey.

Before data collection, this research protocol was reviewed and approved by the Institutional Review Board in A hospital. Full information including research purpose, process, compensation for research participation, voluntary participation and withdrawal from the study were given to all participants, and then written informed consents were received from all participants.

### Data analysis

The data were analyzed using the IBM SPSS 25.0 program (SPSS Inc., Chicago, IL, USA). The normality of the study variables were examined using the Kolmogorov-Smirnov test, with *p* > .05 indicating that the data were normally distributed. Descriptive and inferential statistics (independent t-test and chi-square test) were used to describe and test homogeneity of the participants’ baseline general characteristics and study variables between experimental and control groups. Generalized Estimating Equations (GEE) were used to determine the changes in maternal role confidence and breastfeeding success in time trends (baseline and three more measurement times), differences of variables between groups, and interaction effects between groups and time trends. As post analysis, independent sample t-test was done to verify the differences of the maternal role confidence at each measurement point and the paired t-test was used to verify the differences of study variables amongst four measurement times. ANCOVA analysis was used to verify differences between breastfeeding success scores when adjusting baseline breastfeeding success score.

## Results

### General characteristics of study sample and homogeneity between groups

The 67 mothers ranged in age from 23 to 44 years with a mean of 31 years (standard deviation 4.2). The majority of mothers were university graduates (76.1%), employed (74.6%), in the middle economic class (71.6%) and had given birth vaginally (71.6%). Mean infant birth weight was 3191 g (standard deviation 346.7) ranging from 2500 to 3990 g. When the homogeneity test between control and experimental groups was done, there were no significant differences in all general characteristics (Table 3).

**Table 3:** General characteristics and homogeneity test between two groups (*N* = 67)

Characteristics	Categories	Total (***N*** = 67)	Cont. (***n*** = 37)	Exp. (***n*** = 30)	***χ***^***2***^	***p***
		***n*** (%)	***n*** (%)	***n*** (%)		
Age (years)	< 25	5 (7.4)	2 (5.4)	1 (3.3)	5.88	.118
(M ± SD: 31.0 ± 4.2)	25 ~ 29	21 (31.3)	17 (45.9)	6 (20.0)		
(range: 23 ~ 44)	30 ~ 34	30 (44.8)	14 (37.8)	16 (53.3)		
	≥ 35	11 (16.4)	4 (10.8)	7 (23.3)		
Family type	Couple only	65 (97.0)	35 (94.6)	30 (100.0)	–	.498^†^
	With parents	2 (3.0)	2 (5.4)	0 (0.0)		
Education	High school	6 (9.0)	5 (13.5)	1 (3.3)	2.14	.344
	University	51 (76.1)	27 (73.0)	24 (80.0)		
	Graduate	10 (14.9)	5 (13.5)	5 (16.7)		
Occupation	No	17 (25.4)	18 (48.6)	12 (40.0)	0.50	.479
	Yes	50 (74.6)	19 (51.4)	18 (60.0)		
Economic status	Middle-low	10 (14.9)	6 (16.2)	4 (13.3)	0.76	.684
	Middle	48 (71.6)	25 (67.6)	23 (76.7)		
	Middle-high	9 (13.4)	6 (16.2)	3 (10.0)		
Delivery type	NSVD	48 (71.6)	23 (62.2)	25 (83.3)	3.66	.056
	C-sec	19 (28.4)	14 (37.8)	5 (16.7)		
Baby’s gender	Male	33 (49.3)	9 (42.9)	20 (58.8)	1.33	.249
	Female	34 (50.7)	12 (57.1)	14 (41.2)		
Birth weight (gm)	2500-2999	18 (26.9)	10 (27.0)	8 (26.7)	1.19	.552
(M ± SD: 3191.5 ± 346.7)	3000-3499	35 (52.2)	21 (56.8)	14 (46.7)		
(range: 2500 ~ 3990)	≥3500	14 (20.9)	6 (16.2)	8 (26.7)		

*Cont* Control group, *Exp* Experimental group, *M* Mean, SD Standard Deviation, *NSVD* Normal Spontaneous Vaginal Delivery, *C-sec* Cesarean section

^†^ Fisher’s exact *p*

### Effects of the maternal role adjustment program on maternal role confidence

**Fig. 2:**
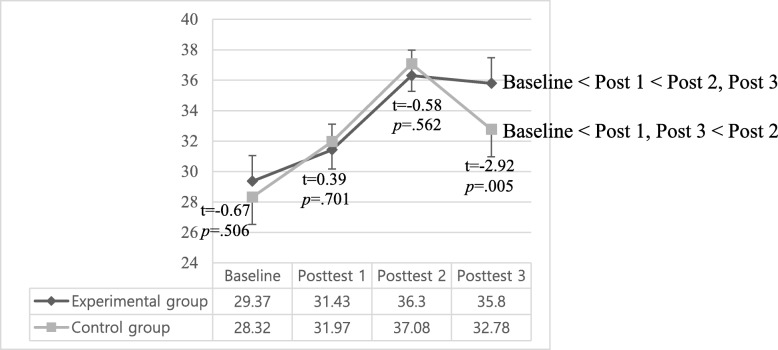
Mean score changes of maternal role confidence. Baseline: 2–7 day after childbirth (the admission day to *Sanhujoriwon*), Posttest1: 2–3 weeks after childbirth (the discharge day from *Sanhujoriwon*), Posttest2: 4–6 weeks after childbirth, Posttest3: 12 weeks after childbirth.

There were significant interaction effects (group x time effect) (*p* = .025) and time effects (*p* < .001) in maternal role confidence. In detail, the scores (mean ± standard deviation) of maternal role confidence in experimental group gradually increased from baseline (29.37 ± 6.59) to posttest1 (31.43 ± 5.12) and posttest2 (36.30 ± 5.36). This increase was maintained to posttest3 (35.80 ± 3.71). While, the scores (mean ± standard deviation) of the control group also increased from baseline (28.32 ± 6.14) to posttest 1 (31.97 ± 6.13) and posttest 2 (37.08 ± 5.52), they dropped significantly at posttest 3 (32.78 ± 4.57). So, maternal role confidence scores at posttest 3 (i.e., 12 weeks postpartum) in the experimental group was higher than that of the control group (t = − 2.92, *p* = .005). In addition, time effect among the four measurement times was significant in each group (*p* < .001) (Table 4, Fig. 2).

**Table 4:** Effects of the maternal role adaptation program (*N* = 67)

Factors	Group	*n*	Baseline M (SD)	Posttest 1 M (SD)	Posttest 2 M (SD)	Posttest 3 M (SD)	*Group* *p*	*Time* *p*	Group*Time *p*
Maternal role confidence	Cont.	37	28.32 (6.14)	31.97 (6.13)	37.08 (5.52)	32.78 (4.57)	.208	<.001	.025
	Exp.	30	29.37 (6.59)	31.43 (5.12)	36.30 (5.36)	35.80 (3.71)			
t(*p*)			−0.67 (.506)	0.39 (.701)	0.58 (.562)	−2.92 (.005)			
Breastfeeding success score	Cont. Exp.	37 30	3.73 (0.65) 2.57 (0.77)	3.76 (0.86) 3.00 (0.98)	3.78 (1.34) 3.73 (1.28)	3.14 (1.87) 3.93 (1.51)	<.001	<.001	<.001
t(*p*) or F^a^(*p*)			6.86 (<.001)	1.43^a^ (.236)	7.97^a^ (.006)	14.31^a^ (<.001)			

Baseline: 2–7 days after childbirth (the admission day to *Sanhujoriwon),* Posttest 1: 2–3 weeks after childbirth (the discharge day from *Sanhujoriwon)*, Posttest 2: 4–6 weeks after childbirth, Posttest 3: 12 weeks after childbirth;

*Cont.* Control group, *Exp.* Experimental group, *M* Mean, *SD* Standard Deviation

^a^ANCOVA test controlling for baseline breastfeeding success score

### Effects of the maternal role adjustment program on breastfeeding success

**Fig. 3:**
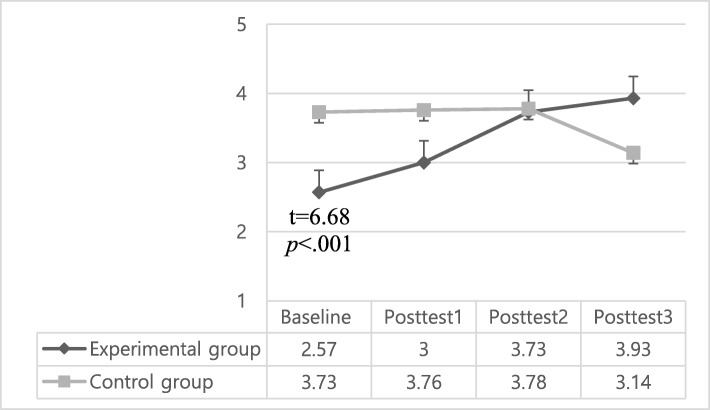
Mean score changes of breastfeeding success score. Baseline: 2–7 day after childbirth (the admission day to *Sanhujoriwon*), Posttest1: 2–3 weeks after childbirth (the discharge day from *Sanhujoriwon*), Posttest2: 4–6 weeks after childbirth, Posttest3: 12 weeks after childbirth.

**Fig. 4:**
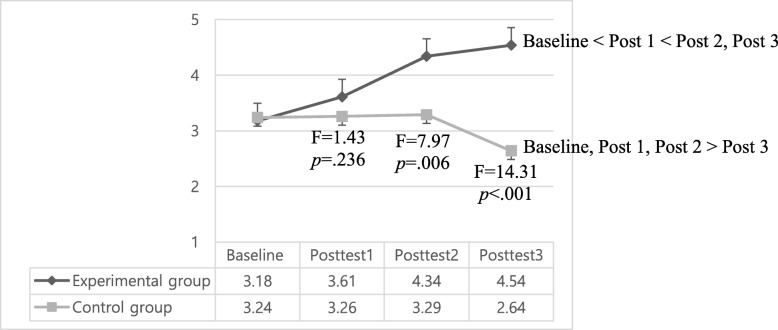
Adjusted mean score changes of breastfeeding success score. Baseline: 2–7 day after childbirth (the admission day to *Sanhujoriwon*), Posttest1: 2–3 weeks after childbirth (the discharge day from *Sanhujoriwon*), Posttest2: 4–6 weeks after childbirth, Posttest3: 12 weeks after childbirth.

In regard to the breastfeeding success scores, there were significant interaction effects (group x time effect) (*p* < .001), time effects (*p* < .001), and group effects (*p* < .001) when controlling for baseline breastfeeding scores. In detail, the breastfeeding success scores (mean ± standard deviation) in the experimental group gradually increased from baseline (2.57 ± 0.77) to posttest 1 (3.00 ± 0.98), posttest 2 (3.73 ± 1.28), and posttest 3 (3.93 ± 1.51). While, the scores (mean ± standard deviation) of the control group showed similar levels from baseline (3.73 ± 0.65) to posttest 1 (3.76 ± 0.86) and posttest 2 (3.78 ± 1.34), but significantly dropped at posttest 3 (3.14 ± 1.87) (Table 4, Fig. 3). So, the experimental group’s breastfeeding success scores were significantly higher than those of control group at both posttest 2 (i.e., 4–6 weeks postpartum) (F = 7.97, *p* = .006) and posttest 3 (i.e., 12 weeks postpartum) (F = 14.31, *p* < .001). In addition, the time effect among the four measurement times were also significant (*p* < .001) (Table 4, Fig. 4).

## Discussion

This study was conducted to evaluate the effectiveness of a maternal role adjustment program based on the ecological model for first time mothers in *Sanhujoriwon* on maternal role confidence and breastfeeding success.

In this study, maternal role confidence in the experimental group gradually increased from baseline (*Sanhujoriwon* admission day) to posttest 1 (*Sanhujoriwon* discharge day) and 2 (4–6 weeks postpartum), and then maintained to posttest 3 (12 weeks postpartum). While the scores of the control group also increased from baseline to posttest 1 and 2, they dropped significantly at posttest 3. As a result, maternal role confidence in the intervention group was significantly higher than that of the control group at posttest 3.

The increase of maternal confidence in both groups from baseline to posttest 1 and 2 was consistent with the results of Shieh et al. [30] and Shorey et al. [31]. Maternal role confidence is associated with cumulative experiences of infant care [32], so mothers in the control group might also increase their maternal role confidence from repeating the daily care for their infants over time [30].

However, in this study maternal role confidence did not increase in both groups from posttest 2 to posttest 3, which is inconsistent with the result of Shorey et al. [31], who reported that maternal confidence gradually increased from baseline to 6 and 12 weeks postpartum in both groups. In most *Sanhujoriwon,* mothers do not choose rooming-in in order to maximize their physical recovery and most of mothers receive their parents’ help for infant care until 4–6 weeks postpartum period even after discharge from the *Sanhujoriwon* [10]. Thus, the mothers in the control group played a more passive mothering role within a supportive environment until 4–6 weeks postpartum, which may have led mothers to underestimate the real difficulties of the mothering role and overestimate their abilities [10, 33]. After 4–6 weeks postpartum, as mothers began to care for their infants independently, many experienced significant challenges in the mothering role. Mothers who have repeated negative experiences may have less efficacy with parenting and be less satisfied with her role as a parent [33]. Therefore, it could be inferred that maternal role confidence in the control group decreased at 12 weeks postpartum compared to 4–6 weeks postpartum. On the other hand, mothers in the experimental group performed their mothering role more independently while rooming-in due to the intervention of this study in *Sanhujoriwon,* which could have had a positive influence on the mother’s infant care ability and maternal role confidence after 4–6 weeks postpartum.

Previous studies have found that social support is an important factor enhancing maternal confidence [32, 34] and support from the partner is often the main source of social support for mothers [34]. Recent societal changes in South Korea have led to more nuclear families living together, rather than the previous norms of extended families cohabitating. This change means that the partner has become the most important social support person to new mothers during the first 4–6 weeks postpartum in the average Korean family. However, social support from partners is often inadequate in the postpartum period [35]. Even though partners want to participate in postpartum care, they often do not have enough education or experience in postpartum and infant care to provide substantial help [36]. In this intervention study, we involved the partners in infant care education and gave partners the tools to better assist new mothers as their most important source of social support. We believe this strategy had a positive effect on the maternal role confidence maintenance at posttest 3 in the intervention group.

In this study, breastfeeding success score of the intervention group continuously increased from baseline (*Sanhujoriwon* admission day) to posttest 3 (12 weeks postpartum), while those of the control group did not show significant changes from baseline to posttest 2 (4–6 weeks postpartum), and dropped at posttest 3. As a result, breastfeeding success scores of the intervention group were significantly higher than those of the control group at posttest 2 (4–6 weeks postpartum) and posttest 3 (12 weeks postpartum,). The higher breastfeeding success scores of the intervention group at posttest 3 was consistent with the results of Abbass-Dick et al. [37]. Infant feeding is the top concern regarding infant care [10, 38]. Mothers wanted to breastfeed because they perceived breastfeeding to be a natural way of feeding that provides various health benefits for infants and mothers [39]. However, the highest drop-out rate of exclusive breastfeeding is noted in the first month postpartum [40]. Perceived insufficient milk is the main reason for breastfeeding discontinuation worldwide [41–44] and is associated with lack of confidence in breastfeeding and misinterpretation of infant’s behavior such as crying [43].

Many mothers perceive infant crying as a sign of hunger and interpret frequent crying to be due to insufficient milk supply. Insufficient milk supply is more likely to be attributed to maternal perception because it is rare to have actual insufficient milk when breastfeeding on demand [41, 45]. In this study, interventions included face-to-face educations and individual counseling regarding infant behaviors such as crying. A more accurate interpretation of infant behavior and response to that through breastfeeding can help first time mothers continue breastfeeding [46, 47] and the rooming-in environment in this study help mothers to know their baby’s behaviors or cues more effectively [11]. Previous research has shown rooming-in is the most important factor to successful breastfeeding according to the principles of the Baby-Friendly Hospital Initiative [48]. Based on the previous literature, as well as the results in our own work, we believe that encouraging breastfeeding practice within an environment that encourages rooming-in was an effective strategy to improve breastfeeding success scores in the experimental group.

Also, offering breastfeeding educations to fathers likely contributed to the higher level of breastfeeding success in the intervention group compared with the control group. Previous studies have reported that inclusion of fathers in breastfeeding interventions resulted in an increase in breastfeeding duration [8, 37, 49] and maternal breastfeeding self-efficacy [9]. Educated fathers provided more help with breastfeeding [37]. Based on the results of previous research and this study, we believe fathers can provide major social support for breastfeeding and should be involved in breastfeeding interventions and maternal adjustment programs.

### Limitations

This study was conducted in two postpartum care centers, i.e., *Sanhujoriwon* located in one city with a quasi-experimental study design. Since randomization was not achieved, we are unable to show causality of the intervention effect on study variables. Further studies using a randomized controlled trial model with more participants living in more cities are warranted. Nevertheless, the results of this novel study were meaningful, as it is the first maternal role adjustment intervention program within a *Sanhujoriwon* based on the ecological model and the concept of family centered care.

## Conclusion

This study demonstrated the effectiveness of a family-centered care education and counseling intervention encouraging infant care practices and breastfeeding while rooming-in with mothers and partners within postpartum care centers. Approaching the family (mother, partner, and infant) as a unit of care was effective in improving maternal role confidence and breastfeeding success among the first time mothers in two South Korean *Sanhujoriwon.* Therefore, this program for enhancing healthy interaction amongst mothers, partners, and infants within an environment that encourages rooming-in should be actively applied in other postpartum care centers. Future research should be done to evaluate the effectiveness of this intervention using randomized controlled trial methods.

## Data Availability

The datasets used and analyzed during the current study are available from the corresponding author on reasonable request.
